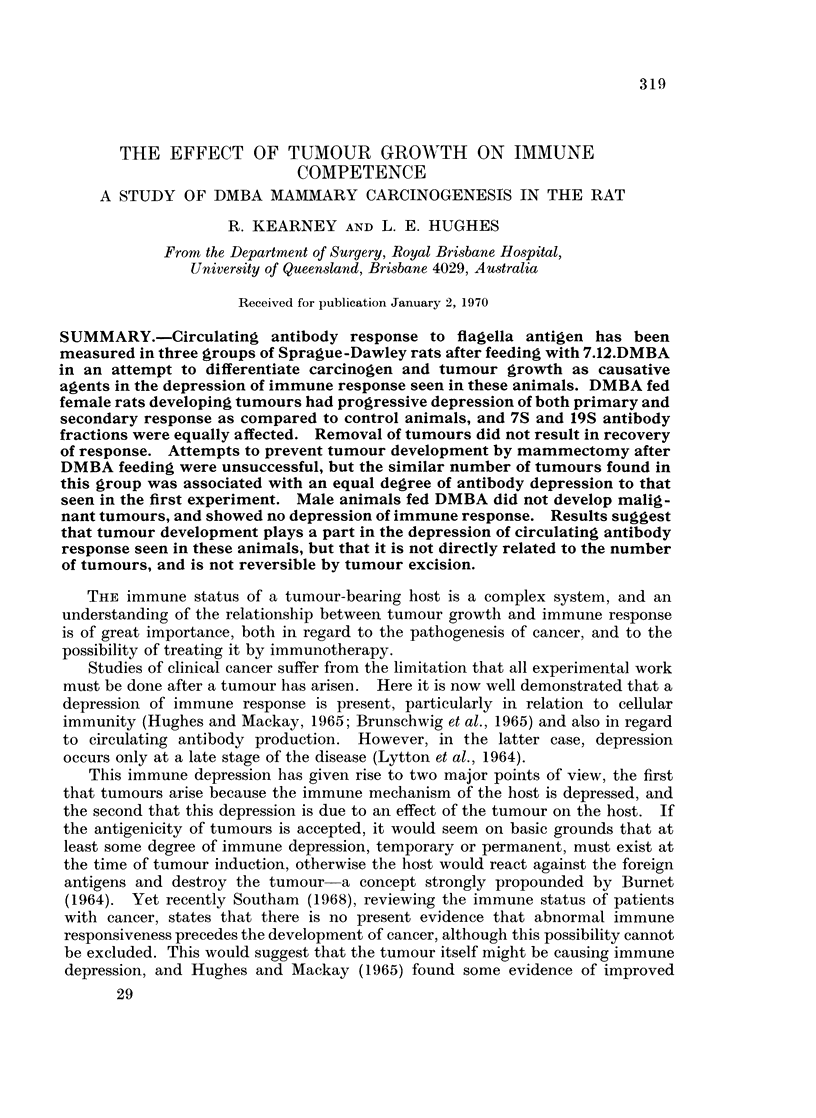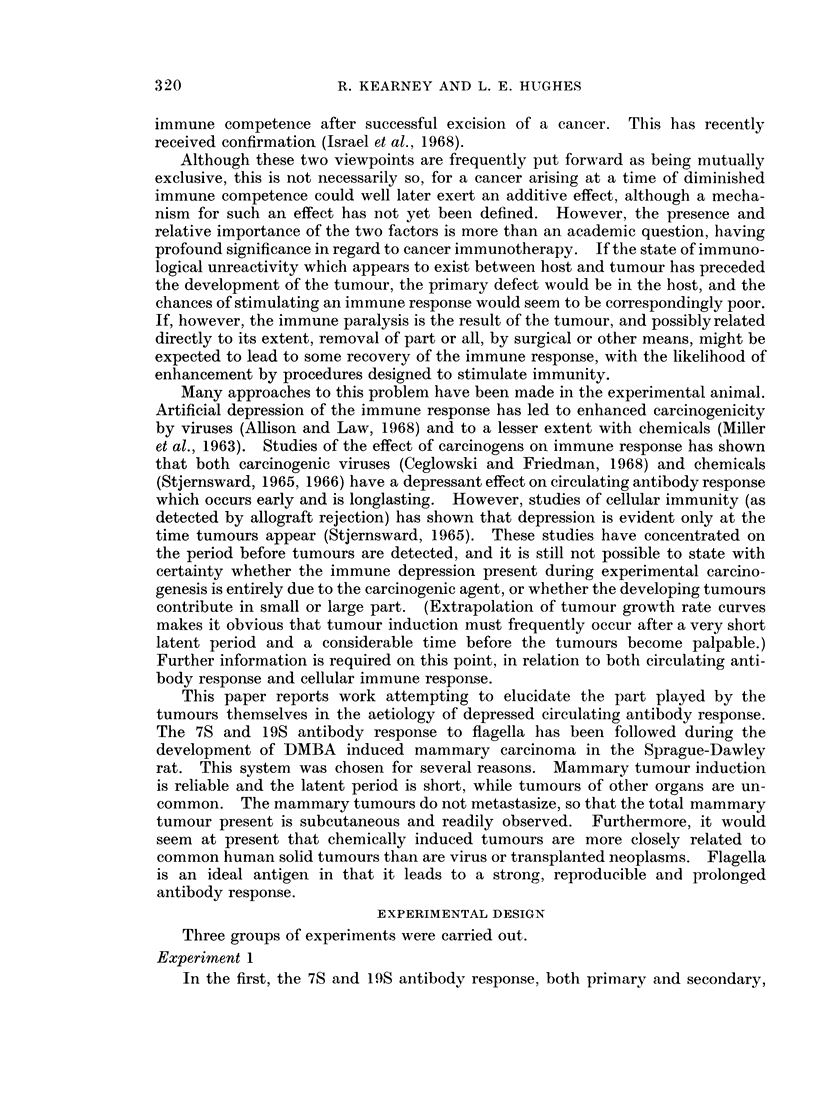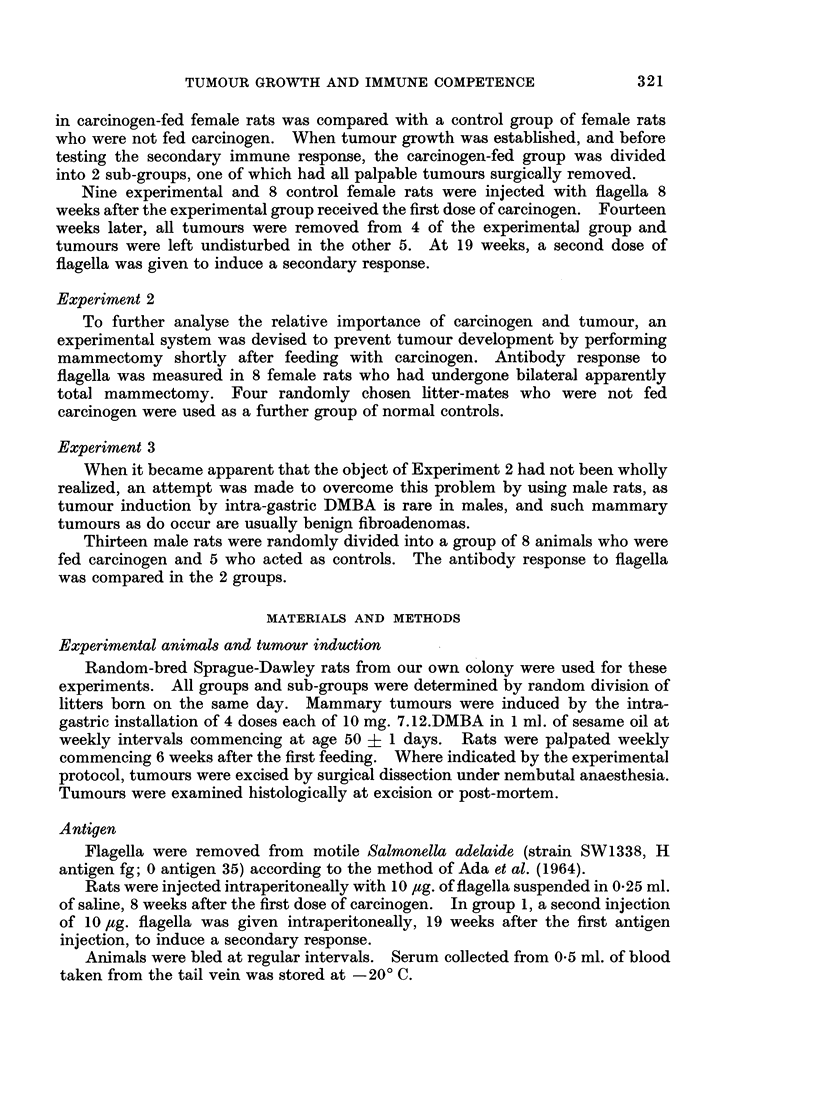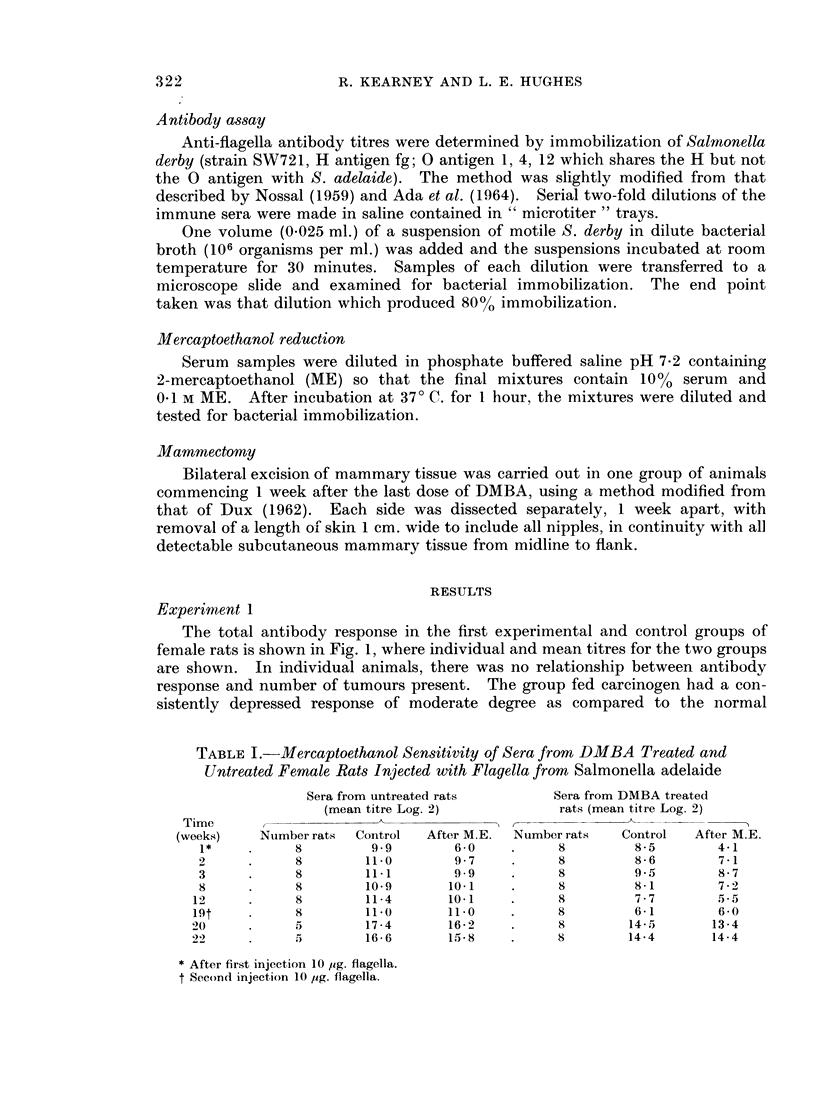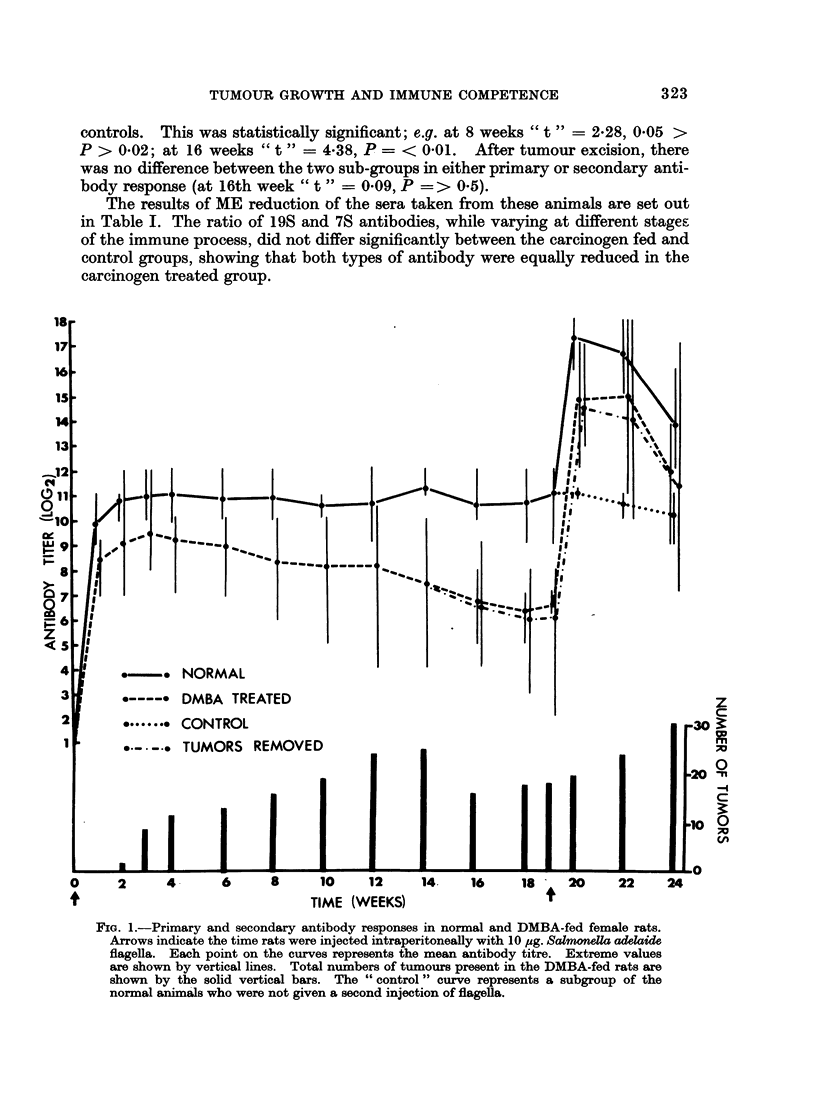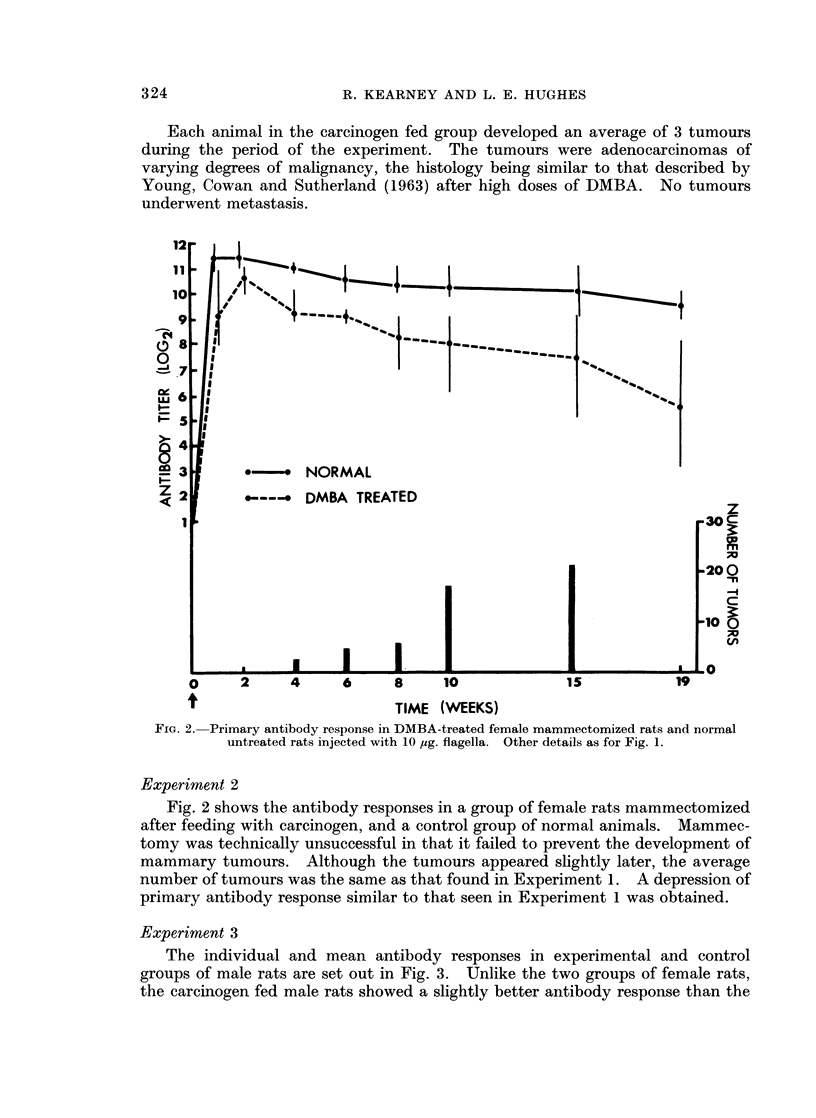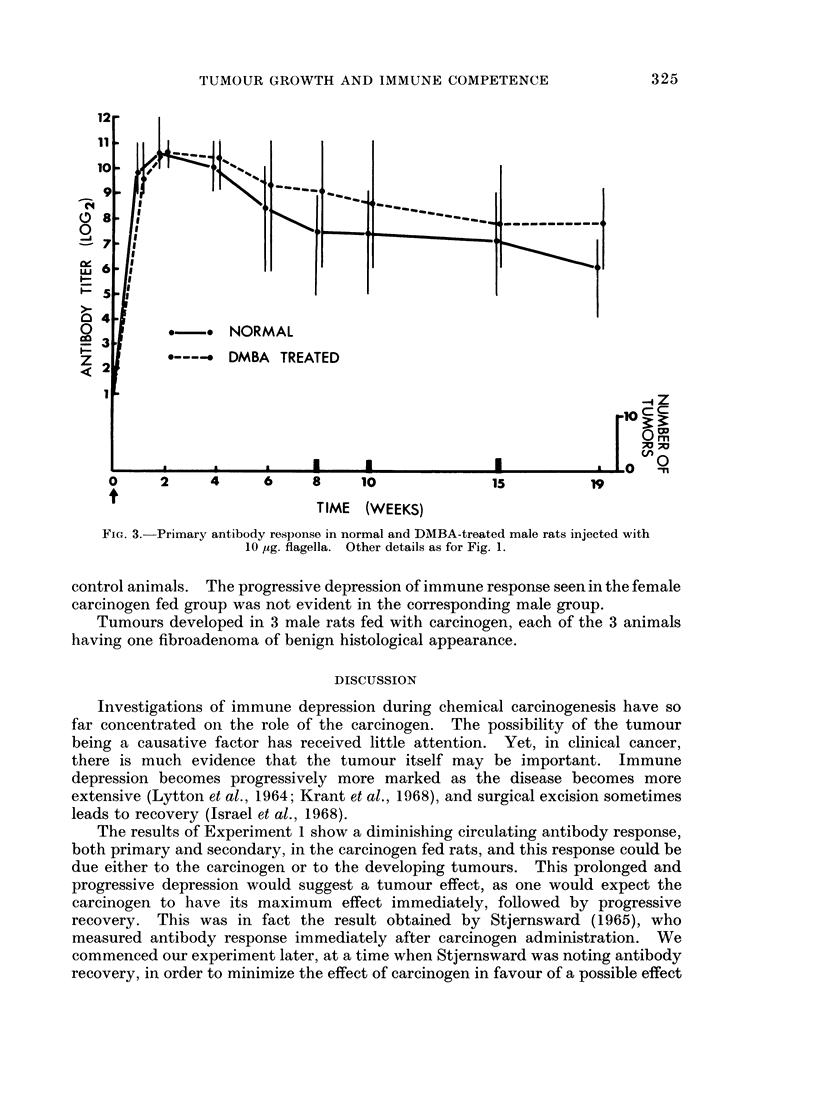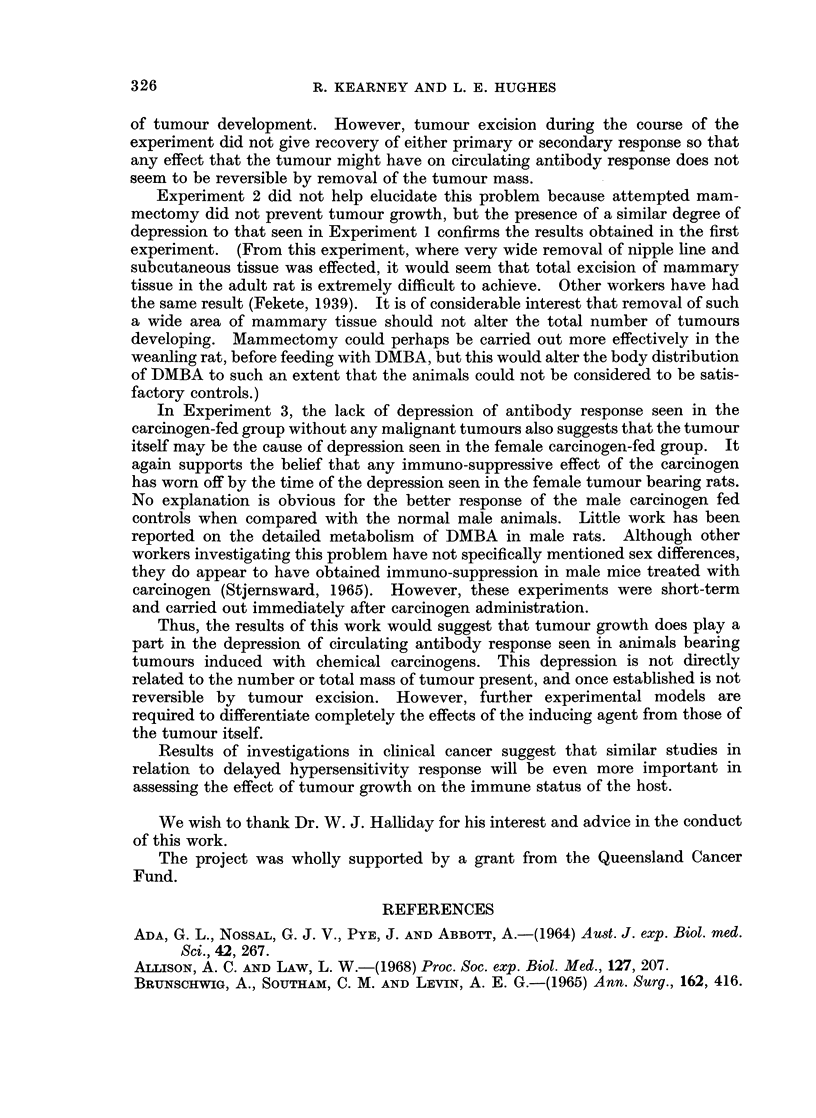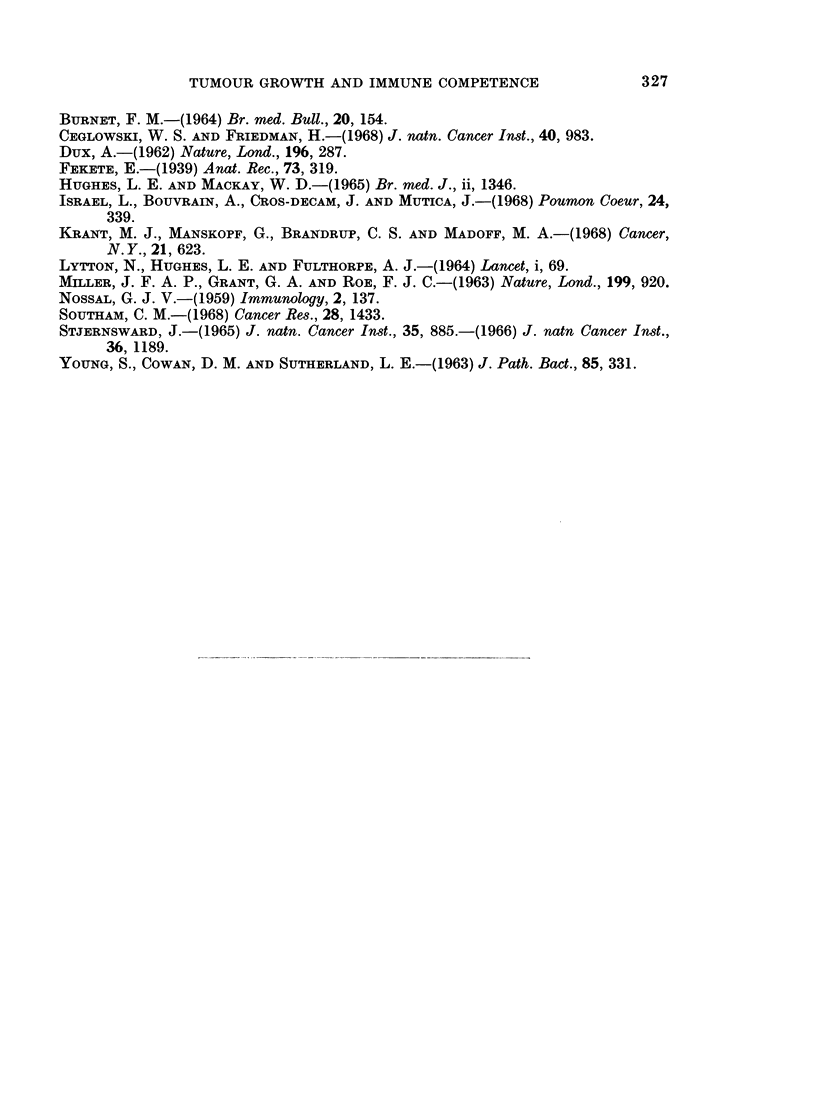# The Effect of Tumour Growth on Immune Competence

**DOI:** 10.1038/bjc.1970.37

**Published:** 1970-06

**Authors:** R. Kearney, L. E. Hughes

## Abstract

Circulating antibody response to flagella antigen has been measured in three groups of Sprague-Dawley rats after feeding with 7.12.DMBA in an attempt to differentiate carcinogen and tumour growth as causative agents in the depression of immune response seen in these animals. DMBA fed female rats developing tumours had progressive depression of both primary and secondary response as compared to control animals, and 7S and 19S antibody fractions were equally affected. Removal of tumours did not result in recovery of response. Attempts to prevent tumour development by mammectomy after DMBA feeding were unsuccessful, but the similar number of tumours found in this group was associated with an equal degree of antibody depression to that seen in the first experiment. Male animals fed DMBA did not develop malignant tumours, and showed no depression of immune response. Results suggest that tumour development plays a part in the depression of circulating antibody response seen in these animals, but that it is not directly related to the number of tumours, and is not reversible by tumour excision.


### THE EFFECT OF TUMOUR GROWTH ON IMMUNE COMPETENCE

### A STUDY OF DMBA MAMMARY CARCINOGENESIS IN THE RAT

R. KEARNEY AND L. E. HUGHES

*From the Department of Surgery, Royal Brisbane Hospital, University of Queensland, Brisbane 4029, Australia*

Received for publication January 2, 1970

**SUMMARY.—Circulating antibody response to flagella antigen has been measured in three groups of Sprague-Dawley rats after feeding with 7.12.DMBA in an attempt to differentiate carcinogen and tumour growth as causative agents in the depression of immune response seen in these animals. DMBA fed female rats developing tumours had progressive depression of both primary and secondary response as compared to control animals, and 7S and 19S antibody fractions were equally affected. Removal of tumours did not result in recovery of response. Attempts to prevent tumour development by mammectomy after DMBA feeding were unsuccessful, but the similar number of tumours found in this group was associated with an equal degree of antibody depression to that seen in the first experiment. Male animals fed DMBA did not develop malignant tumours, and showed no depression of immune response. Results suggest that tumour development plays a part in the depression of circulating antibody response seen in these animals, but that it is not directly related to the number of tumours, and is not reversible by tumour excision.**

THE immune status of a tumour-bearing host is a complex system, and an understanding of the relationship between tumour growth and immune response is of great importance, both in regard to the pathogenesis of cancer, and to the possibility of treating it by immunotherapy.

Studies of clinical cancer suffer from the limitation that all experimental work must be done after a tumour has arisen. Here it is now well demonstrated that a depression of immune response is present, particularly in relation to cellular immunity (Hughes and Mackay, 1965; Brunschwig *et al.*, 1965) and also in regard to circulating antibody production. However, in the latter case, depression occurs only at a late stage of the disease (Lytton *et al.*, 1964).

This immune depression has given rise to two major points of view, the first that tumours arise because the immune mechanism of the host is depressed, and the second that this depression is due to an effect of the tumour on the host. If the antigenicity of tumours is accepted, it would seem on basic grounds that at least some degree of immune depression, temporary or permanent, must exist at the time of tumour induction, otherwise the host would react against the foreign antigens and destroy the tumour—a concept strongly propounded by Burnet (1964). Yet recently Southam (1968), reviewing the immune status of patients with cancer, states that there is no present evidence that abnormal immune responsiveness precedes the development of cancer, although this possibility cannot be excluded. This would suggest that the tumour itself might be causing immune depression, and Hughes and Mackay (1965) found some evidence of improved immune competence after successful excision of a cancer. This has recently received confirmation (Israel *et al.*, 1968).

Although these two viewpoints are frequently put forward as being mutually exclusive, this is not necessarily so, for a cancer arising at a time of diminished immune competence could well later exert an additive effect, although a mechanism for such an effect has not yet been defined. However, the presence and relative importance of the two factors is more than an academic question, having profound significance in regard to cancer immunotherapy. If the state of immunological unreactivity which appears to exist between host and tumour has preceded the development of the tumour, the primary defect would be in the host, and the chances of stimulating an immune response would seem to be correspondingly poor. If, however, the immune paralysis is the result of the tumour, and possibly related directly to its extent, removal of part or all, by surgical or other means, might be expected to lead to some recovery of the immune response, with the likelihood of enhancement by procedures designed to stimulate immunity.

Many approaches to this problem have been made in the experimental animal. Artificial depression of the immune response has led to enhanced carcinogenicity by viruses (Allison and Law, 1968) and to a lesser extent with chemicals (Miller *et al.*, 1963). Studies of the effect of carcinogens on immune response has shown that both carcinogenic viruses (Ceglowski and Friedman, 1968) and chemicals (Stjernsward, 1965, 1966) have a depressant effect on circulating antibody response which occurs early and is longlasting. However, studies of cellular immunity (as detected by allograft rejection) has shown that depression is evident only at the time tumours appear (Stjernsward, 1965). These studies have concentrated on the period before tumours are detected, and it is still not possible to state with certainty whether the immune depression present during experimental carcinogenesis is entirely due to the carcinogenic agent, or whether the developing tumours contribute in small or large part. (Extrapolation of tumour growth rate curves makes it obvious that tumour induction must frequently occur after a very short latent period and a considerable time before the tumours become palpable.) Further information is required on this point, in relation to both circulating antibody response and cellular immune response.

This paper reports work attempting to elucidate the part played by the tumours themselves in the aetiology of depressed circulating antibody response. The 7S and 19S antibody response to flagella has been followed during the development of DMBA induced mammary carcinoma in the Sprague-Dawley rat. This system was chosen for several reasons. Mammary tumour induction is reliable and the latent period is short, while tumours of other organs are uncommon. The mammary tumours do not metastasize, so that the total mammary tumour present is subcutaneous and readily observed. Furthermore, it would seem at present that chemically induced tumours are more closely related to common human solid tumours than are virus or transplanted neoplasms. Flagella is an ideal antigen in that it leads to a strong, reproducible and prolonged antibody response.

### EXPERIMENTAL DESIGN

Three groups of experiments were carried out.

### *Experiment* 1

In the first, the 7S and 19S antibody response, both primary and secondary, in carcinogen-fed female rats was compared with a control group of female rats who were not fed carcinogen. When tumour growth was established, and before testing the secondary immune response, the carcinogen-fed group was divided into 2 sub-groups, one of which had all palpable tumours surgically removed.

Nine experimental and 8 control female rats were injected with flagella 8 weeks after the experimental group received the first dose of carcinogen. Fourteen weeks later, all tumours were removed from 4 of the experimental group and tumours were left undisturbed in the other 5. At 19 weeks, a second dose of flagella was given to induce a secondary response.

*Experiment* 2

To further analyse the relative importance of carcinogen and tumour, an experimental system was devised to prevent tumour development by performing mammectomy shortly after feeding with carcinogen. Antibody response to flagella was measured in 8 female rats who had undergone bilateral apparently total mammectomy. Four randomly chosen litter-mates who were not fed carcinogen were used as a further group of normal controls.

*Experiment* 3

When it became apparent that the object of Experiment 2 had not been wholly realized, an attempt was made to overcome this problem by using male rats, as tumour induction by intra-gastric DMBA is rare in males, and such mammary tumours as do occur are usually benign fibroadenomas.

Thirteen male rats were randomly divided into a group of 8 animals who were fed carcinogen and 5 who acted as controls. The antibody response to flagella was compared in the 2 groups.

### MATERIALS AND METHODS

*Experimental animals and tumour induction*

Random-bred Sprague-Dawley rats from our own colony were used for these experiments. All groups and sub-groups were determined by random division of litters born on the same day. Mammary tumours were induced by the intra-gastric installation of 4 doses each of 10 mg. 7.12.DMBA in 1 ml. of sesame oil at weekly intervals commencing at age $50 \pm 1$ days. Rats were palpated weekly commencing 6 weeks after the first feeding. Where indicated by the experimental protocol, tumours were excised by surgical dissection under nembutal anaesthesia. Tumours were examined histologically at excision or post-mortem.

*Antigen*

Flagella were removed from motile *Salmonella adelaide* (strain SW1338, H antigen fg; 0 antigen 35) according to the method of Ada *et al.* (1964).

Rats were injected intraperitoneally with 10 $\mu$g. of flagella suspended in 0·25 ml. of saline, 8 weeks after the first dose of carcinogen. In group 1, a second injection of 10 $\mu$g. flagella was given intraperitoneally, 19 weeks after the first antigen injection, to induce a secondary response.

Animals were bled at regular intervals. Serum collected from 0·5 ml. of blood taken from the tail vein was stored at $-20°$ C.

*Antibody assay*

Anti-flagella antibody titres were determined by immobilization of *Salmonella derby* (strain SW721, H antigen fg; O antigen 1, 4, 12 which shares the H but not the O antigen with *S. adelaide*). The method was slightly modified from that described by Nossal (1959) and Ada *et al.* (1964). Serial two-fold dilutions of the immune sera were made in saline contained in " microtiter " trays.

One volume (0·025 ml.) of a suspension of motile *S. derby* in dilute bacterial broth ($10^6$ organisms per ml.) was added and the suspensions incubated at room temperature for 30 minutes. Samples of each dilution were transferred to a microscope slide and examined for bacterial immobilization. The end point taken was that dilution which produced 80% immobilization.

*Mercaptoethanol reduction*

Serum samples were diluted in phosphate buffered saline pH 7·2 containing 2-mercaptoethanol (ME) so that the final mixtures contain 10% serum and 0·1 M ME. After incubation at 37° C. for 1 hour, the mixtures were diluted and tested for bacterial immobilization.

*Mammectomy*

Bilateral excision of mammary tissue was carried out in one group of animals commencing 1 week after the last dose of DMBA, using a method modified from that of Dux (1962). Each side was dissected separately, 1 week apart, with removal of a length of skin 1 cm. wide to include all nipples, in continuity with all detectable subcutaneous mammary tissue from midline to flank.

### RESULTS

*Experiment* 1

The total antibody response in the first experimental and control groups of female rats is shown in Fig. 1, where individual and mean titres for the two groups are shown. In individual animals, there was no relationship between antibody response and number of tumours present. The group fed carcinogen had a consistently depressed response of moderate degree as compared to the normal

TABLE I.—*Mercaptoethanol Sensitivity of Sera from DMBA Treated and Untreated Female Rats Injected with Flagella from* Salmonella adelaide

| Time (weeks) | Sera from untreated rats (mean titre Log. 2) | | | Sera from DMBA treated rats (mean titre Log. 2) | | |
|---|---|---|---|---|---|---|
| | Number rats | Control | After M.E. | Number rats | Control | After M.E. |
| 1* | 8 | 9·9 | 6·0 | 8 | 8·5 | 4·1 |
| 2 | 8 | 11·0 | 9·7 | 8 | 8·6 | 7·1 |
| 3 | 8 | 11·1 | 9·9 | 8 | 9·5 | 8·7 |
| 8 | 8 | 10·9 | 10·1 | 8 | 8·1 | 7·2 |
| 12 | 8 | 11·4 | 10·1 | 8 | 7·7 | 5·5 |
| 19† | 8 | 11·0 | 11·0 | 8 | 6·1 | 6·0 |
| 20 | 5 | 17·4 | 16·2 | 8 | 14·5 | 13·4 |
| 22 | 5 | 16·6 | 15·8 | 8 | 14·4 | 14·4 |

* After first injection 10 μg. flagella.

† Second injection 10 μg. flagella.

controls. This was statistically significant; *e.g.* at 8 weeks " t " = 2·28, 0·05 > $P$ > 0·02; at 16 weeks " t " = 4·38, $P$ = < 0·01. After tumour excision, there was no difference between the two sub-groups in either primary or secondary antibody response (at 16th week " t " = 0·09, $P$ => 0·5).

The results of ME reduction of the sera taken from these animals are set out in Table I. The ratio of 19S and 7S antibodies, while varying at different stages of the immune process, did not differ significantly between the carcinogen fed and control groups, showing that both types of antibody were equally reduced in the carcinogen treated group.

FIG. 1.—Primary and secondary antibody responses in normal and DMBA-fed female rats. Arrows indicate the time rats were injected intraperitoneally with 10 μg. *Salmonella adelaide* flagella. Each point on the curves represents the mean antibody titre. Extreme values are shown by vertical lines. Total numbers of tumours present in the DMBA-fed rats are shown by the solid vertical bars. The " control " curve represents a subgroup of the normal animals who were not given a second injection of flagella.

Each animal in the carcinogen fed group developed an average of 3 tumours during the period of the experiment. The tumours were adenocarcinomas of varying degrees of malignancy, the histology being similar to that described by Young, Cowan and Sutherland (1963) after high doses of DMBA. No tumours underwent metastasis.

Fig. 2.—Primary antibody response in DMBA-treated female mammectomized rats and normal untreated rats injected with 10 μg. flagella. Other details as for Fig. 1.

*Experiment* 2

Fig. 2 shows the antibody responses in a group of female rats mammectomized after feeding with carcinogen, and a control group of normal animals. Mammectomy was technically unsuccessful in that it failed to prevent the development of mammary tumours. Although the tumours appeared slightly later, the average number of tumours was the same as that found in Experiment 1. A depression of primary antibody response similar to that seen in Experiment 1 was obtained.

*Experiment* 3

The individual and mean antibody responses in experimental and control groups of male rats are set out in Fig. 3. Unlike the two groups of female rats, the carcinogen fed male rats showed a slightly better antibody response than the

FIG. 3.—Primary antibody response in normal and DMBA-treated male rats injected with 10 μg. flagella. Other details as for Fig. 1.

control animals. The progressive depression of immune response seen in the female carcinogen fed group was not evident in the corresponding male group.

Tumours developed in 3 male rats fed with carcinogen, each of the 3 animals having one fibroadenoma of benign histological appearance.

### DISCUSSION

Investigations of immune depression during chemical carcinogenesis have so far concentrated on the role of the carcinogen. The possibility of the tumour being a causative factor has received little attention. Yet, in clinical cancer, there is much evidence that the tumour itself may be important. Immune depression becomes progressively more marked as the disease becomes more extensive (Lytton *et al.*, 1964; Krant *et al.*, 1968), and surgical excision sometimes leads to recovery (Israel *et al.*, 1968).

The results of Experiment 1 show a diminishing circulating antibody response, both primary and secondary, in the carcinogen fed rats, and this response could be due either to the carcinogen or to the developing tumours. This prolonged and progressive depression would suggest a tumour effect, as one would expect the carcinogen to have its maximum effect immediately, followed by progressive recovery. This was in fact the result obtained by Stjernsward (1965), who measured antibody response immediately after carcinogen administration. We commenced our experiment later, at a time when Stjernsward was noting antibody recovery, in order to minimize the effect of carcinogen in favour of a possible effect of tumour development. However, tumour excision during the course of the experiment did not give recovery of either primary or secondary response so that any effect that the tumour might have on circulating antibody response does not seem to be reversible by removal of the tumour mass.

Experiment 2 did not help elucidate this problem because attempted mammectomy did not prevent tumour growth, but the presence of a similar degree of depression to that seen in Experiment 1 confirms the results obtained in the first experiment. (From this experiment, where very wide removal of nipple line and subcutaneous tissue was effected, it would seem that total excision of mammary tissue in the adult rat is extremely difficult to achieve. Other workers have had the same result (Fekete, 1939). It is of considerable interest that removal of such a wide area of mammary tissue should not alter the total number of tumours developing. Mammectomy could perhaps be carried out more effectively in the weanling rat, before feeding with DMBA, but this would alter the body distribution of DMBA to such an extent that the animals could not be considered to be satisfactory controls.)

In Experiment 3, the lack of depression of antibody response seen in the carcinogen-fed group without any malignant tumours also suggests that the tumour itself may be the cause of depression seen in the female carcinogen-fed group. It again supports the belief that any immuno-suppressive effect of the carcinogen has worn off by the time of the depression seen in the female tumour bearing rats. No explanation is obvious for the better response of the male carcinogen fed controls when compared with the normal male animals. Little work has been reported on the detailed metabolism of DMBA in male rats. Although other workers investigating this problem have not specifically mentioned sex differences, they do appear to have obtained immuno-suppression in male mice treated with carcinogen (Stjernsward, 1965). However, these experiments were short-term and carried out immediately after carcinogen administration.

Thus, the results of this work would suggest that tumour growth does play a part in the depression of circulating antibody response seen in animals bearing tumours induced with chemical carcinogens. This depression is not directly related to the number or total mass of tumour present, and once established is not reversible by tumour excision. However, further experimental models are required to differentiate completely the effects of the inducing agent from those of the tumour itself.

Results of investigations in clinical cancer suggest that similar studies in relation to delayed hypersensitivity response will be even more important in assessing the effect of tumour growth on the immune status of the host.

We wish to thank Dr. W. J. Halliday for his interest and advice in the conduct of this work.

The project was wholly supported by a grant from the Queensland Cancer Fund.

### REFERENCES

Ada, G. L., Nossal, G. J. V., Pye, J. and Abbott, A.—(1964) *Aust. J. exp. Biol. med. Sci.*, **42**, 267.

Allison, A. C. and Law, L. W.—(1968) *Proc. Soc. exp. Biol. Med.*, **127**, 207.

Brunschwig, A., Southam, C. M. and Levin, A. E. G.—(1965) *Ann. Surg.*, **162**, 416.

BURNET, F. M.—(1964) *Br. med. Bull.*, **20**, 154.
CEGLOWSKI, W. S. AND FRIEDMAN, H.—(1968) *J. natn. Cancer Inst.*, **40**, 983.
DUX, A.—(1962) *Nature, Lond.*, **196**, 287.
FEKETE, E.—(1939) *Anat. Rec.*, **73**, 319.
HUGHES, L. E. AND MACKAY, W. D.—(1965) *Br. med. J.*, ii, 1346.
ISRAEL, L., BOUVRAIN, A., CROS-DECAM, J. AND MUTICA, J.—(1968) *Poumon Coeur*, **24**, 339.
KRANT, M. J., MANSKOPF, G., BRANDRUP, C. S. AND MADOFF, M. A.—(1968) *Cancer, N.Y.*, **21**, 623.
LYTTON, N., HUGHES, L. E. AND FULTHORPE, A. J.—(1964) *Lancet*, i, 69.
MILLER, J. F. A. P., GRANT, G. A. AND ROE, F. J. C.—(1963) *Nature, Lond.*, **199**, 920.
NOSSAL, G. J. V.—(1959) *Immunology*, **2**, 137.
SOUTHAM, C. M.—(1968) *Cancer Res.*, **28**, 1433.
STJERNSWARD, J.—(1965) *J. natn. Cancer Inst.*, **35**, 885.—(1966) *J. natn Cancer Inst.*, **36**, 1189.
YOUNG, S., COWAN, D. M. AND SUTHERLAND, L. E.—(1963) *J. Path. Bact.*, **85**, 331.